# An Integrated Strategy for Autonomous Exploration of Spatial Processes in Unknown Environments

**DOI:** 10.3390/s20133663

**Published:** 2020-06-30

**Authors:** Valentina Karolj, Alberto Viseras, Luis Merino, Dmitriy Shutin

**Affiliations:** 1 Service Robotics Laboratory, Universidad Pablo de Olavide, Crta. Utrera km 1, 41013 Seville, Spain; lmercab@upo.es; 2 German Aerospace Center, Oberpfaffenhofen, 82234 Weßling, Germany; alberto.viserasruiz@dlr.de (A.V.); dmitriy.shutin@dlr.de (D.S.)

**Keywords:** Gaussian process, exploration, mapping, autonomous robots, mobile robots, information gathering

## Abstract

Exploration of spatial processes, such as radioactivity or temperature is a fundamental task in many robotic applications. In the literature, robotic exploration is mainly carried out for applications where the environment is a priori known. However, for most real life applications this assumption often does not hold, specifically for disaster scenarios. In this paper, we propose a novel integrated strategy that allows a robot to explore a spatial process of interest in an unknown environment. To this end, we build upon two major blocks. First, we propose the use of GP to model the spatial process of interest, and process entropy to drive the exploration. Second, we employ registration algorithms for robot mapping and localization, and frontier-based exploration to explore the environment. However, map and process exploration can be conflicting goals. Our integrated strategy fuses the two aforementioned blocks through a trade-off between process and map exploration. We carry out extensive evaluations of our algorithm in simulated environments with respect to different baselines and environment setups using simulated GP data as a process at hand. Additionally, we perform experimental verification with a mobile holonomic robot exploring a simulated process in an unknown labyrinth environment. Demonstrated results show that our integrated strategy outperforms both frontier-based and GP entropy-driven exploration strategies.

## 1. Introduction

### 1.1. Motivation

Autonomous robots are well suited for gathering information in hostile or hardly accessible environments. For instance, they can be used to map radiation or gas concentration levels in NBC hazards [1,2], temperature and/or humidity fields, etc. If the environment is partially or fully unknown, a robot needs to explore and build a 2D/3D obstacle map to ensure that the whole environment is covered. At the same time, it needs to safely navigate through the operating environment and localize itself and the gathered information with respect to such a map in the absence of global localization.

Over the last few decades, mapping and SLAM have been areas of extensive research in the robotics community for obtaining accurate maps of unknown environments [3]. Moreover, the extension to active mapping and active SLAM has been considered [4,5]. While active mapping determines the actions of the robot that improve area coverage [6], active SLAM considers at the same time maximization of coverage while maintaining localization performance [7].

In many cases, active mapping and active SLAM are required for proper localization of the robot, construction of an accurate map, and navigation purposes. These are essential support for the main mission of the robot, e.g., monitoring radiation, magnetic field, temperature distribution, etc. The main problem considered here is how a robot can efficiently carry out this main mission (explore such fields) while building at the same time the map of the environment.

Ideas similar to active mapping can be used to explore the spatial field [8]. However, this can lead to potentially conflicting goals, given that different models and sensors (with a different FOV, etc.) are used for building the 2D/3D obstacle map and for field reconstruction. This paper thus deals with developing techniques for autonomous exploration of such processes in completely unknown environments, which consider at the same time and in an integrated way efficient map building and process estimation. We focus on exploring physical processes that can be modelled as GP [9]. Examples of such a process are temperature, ozone concentration, and magnetic field intensity, to name only a few.

We challenge here two fundamental assumptions that are typically considered in the state of the art: (i) A priori knowledge about the operating environment (the map), and (ii) that process and map exploration can be solved independently. The aim of our integrated strategy is to minimize the error in the process reconstruction and to maximize the coverage of the environment map, while minimizing the total travelled distance of the exploratory mission all together.

### 1.2. Related Work

Recent advances in mobile robotics have opened new frontiers for the development of novel exploration algorithms. In the literature, approaches for robot exploration are typically based on the maximum informativeness criterion (e.g., [8,10,11,12]), which guides the robot towards locations with the highest information gain.

We are interested in complex probabilistic models that allow us to model environmental state and learn the process from acquired data while exploiting the spatial correlation between variables of the process in order to obtain faster exploration results. GPs represent a powerful approach to model spatial phenomena. Singh et al. [11] propose a procedure to define suitable covariance functions for Gaussian process regression in environmental surveillance applications. Then they extend those covariance functions to perform informative sampling often combined with GPs due to their ability to predict the remaining uncertainty about the process.

Krause et al. study what is the optimal placement for a network of sensors in order to reduce the uncertainty of the GP [10]. They consider the sensor placements as fixed. In contrast, we are interested in exploration with a mobile robot. This allows us to cover larger environments, gives us more flexibility to monitor dynamic processes, and re-plan in newly discovered areas.

Informative sampling that takes into account an information metric (i.e., mutual information that takes into account statistical dependencies between sampling point candidates) in combination with GPs has been considered for different exploration applications, e.g., magnetic field mapping [8], environmental monitoring [13], and online estimation of a radio signal source [14]. However, the works in [8,13,14] assume the environment in which the robot operates as a priori known. In order to deploy autonomous robots in complex environments, we must develop algorithms that are able to operate in unknown environments.

Active SLAM approaches tackle the operation in unknown environments while considering joint uncertainty reduction for map and robot pose trajectory. Common frameworks consider information theoretical approaches where decision is based on the notion of information gain, guiding the robot towards locations where information about unknown environment can be obtained [5,15]. Occupancy grids are commonly used for map representation and navigation purposes [16]. Besides occupancy grids, GPs are introduced as continuous parametrization of frontiers for autonomous exploration in combination with SLAM [17]. There, the authors use BCM to incrementally build GP maps of the environment while guiding the robot toward borders with unknown regions on the map. However, the aforementioned approaches do not incorporate the exploration of a spatial process within the framework of a geometrical map exploration.

Approaches, where SLAM and a spatially distributed process are jointly considered, are exemplified in [18]. There, the authors describe a gas distribution mapping algorithm which is able to consider and propagate the uncertainty in the pose estimate, integrating it with SLAM to create two independent occupancy grids for (i) environment and (ii) gas distribution. However, the exploration strategy is not considered, and the system is teleoperated.

Besides occupancy maps, other spatial processes could be used as anchors for geometric SLAM. For instance, Jung et al. [19] introduce magnetic field constraints for pose-SLAM optimization for loop-closure and heading correction with off-line processing of the gathered magnetic sensor data for creation of the pose-SLAM. As in the previous case, active mapping component is not considered.

Prágr et al. [20] propose a greedy approach that combines spatial exploration (mapping) with the exploration of the underlying traversal cost model over terrain by employing the RBCM. Their approach is similar to ours with respect to the combination of GP and frontier-based exploration. However, a key difference is that we consider a GP that is not necessarily correlated with the spatial map—by mapping new regions of the environment via perception sensor, we do not gain additional information about the process like in their case. With GP reconstruction, useful knowledge of the process increases only in the vicinity where measurements are obtained. A second key difference is that, in contrast to the aforementioned greedy strategy [20], we present a multi-step horizon planning for the integrated exploration that takes into account spatial field exploration on a smaller scale than the environment mapping.

An interesting approach to the multi-step-ahead exploration strategy is the use of TSP to model the goal selection problem. Travelled distance can be described as a cost to be minimized while visiting all goals. Kulich et al. [21] introduce a TSP-based approach for finding a minimum distance path to all frontiers instead of greedily visiting frontiers one by one, benefitting in a possible shorter exploration time. Oßwald et al. [22] apply a similar strategy by exploiting prior knowledge and running a TSP on an user-defined topological graph. They combine this solution with local exploration of individual locations and the results demonstrate a significant reduction in exploration time. Authors in [23] apply constrained generalized TSP to solve map coverage while integrating goal selection with the goal candidates generation. In this work, we apply a similar method for minimizing distance in several steps of our exploration strategy.

In contrast to map coverage in [21,22,23], our focus is on the joint exploration of map and GP. The exploration objectives include a multi-step exploration strategy, considering that the map has to be fully covered, but at the same time the process exploration task has to be fulfilled. This can be viewed as a specific case of a multi-objective optimization for the most informative candidate points selection that are (i) extending map coverage, (ii) decreasing process error.

There has been extensive work on multi-objective approaches that combine exploration in unknown environments and mapping [24,25,26]. Classical approaches towards a solution to this complex problem consider various perspectives e.g., (i) aggregation into a single utility function [15,24,27]; (ii) Pareto efficiency, i.e., improving one objective will negatively affect the others [25]; (iii) domain specific robot exploration and search problem represented as a high-level state machine—Petri nets [26]. Aggregation of different criteria (i.e., distance, information gain, localization uncertainty, communication, etc.) has mostly ad hoc design due to sensitivity to combined criteria, and it requires careful weight prioritisation. Stachniss and Burgard [27] apply a weight tuning to combine the uncertainty reduction with the distance travelled. Carrillo et al. [15] introduce an automatically tuned factor for a trade-off in exploration–exploitation dilemma in SLAM. Despite overcoming weight set-up, their approach cannot be generalized to process exploration—it is task specific for combining localization and mapping. A more general multi-criteria approach is proposed as aggregation operator obtained from Choquet integral [28], and weighted means by Basilico and Amigoni [24]. They apply multi-criteria decision making theoretical approach for searching fixed targets (victims) in a priori unknown environment. Such an approach offers more flexibility for composing multi-objective criteria; however it still has the drawback of weight setting. Amigoni and Gallo [25] solve this problem of weighting utilities as a multi-objective optimization by finding a Pareto front as the best trade-off between competing objectives for a multi-robot system. Candidates are Pareto efficient with respect to travelling cost, information gain, and expected overlap between robots. The best position is greedily chosen from the Pareto frontier as the nearest distance from the ideal solution (i.e., best travelling cost, best information gain, and best overlap). Their approach to find a good simultaneous solution for all objectives is interesting, however it does not guarantee that the chosen candidate is the best among Pareto efficient candidates. A more intuitive, high level representation of multi-objective optimization for exploration and search strategies is presented by Calisi et al. [26]. They represent robot plans as Petri nets, adapted to the tasks of exploration, search and rescue missions. Petri nets offer a modular approach in resolving complex situations such as loops, interrupts, etc. Authors apply it for map exploration while assuming that interesting features can be always intercepted by the same sensor used for mapping. With that assumption, it is only needed to search for victims along the obstacles detected by the laser scanner while avoiding unnecessary search in open space. To the contrary with Calisi et al. [26], we need to explicitly go into open space since the obstacle perception sensor does not provide us knowledge about the process, which is collected with a point-wise sensor.

To the best of our knowledge, there is a research gap in combining mapping and process exploration strategies. Particularly in cases where, given sensors on a different scale, obtained data from perception sensor does not produce any new information about the underlying spatial process we want to explore.

### 1.3. Contribution

The main contribution of this paper is a novel integrated strategy for map and physical process exploration in an unknown environment with the following characteristics:

- It considers multi-step exploration, which has proven to be superior in comparison with greedy, one-step strategies [21,22].
- It selects intermediate goals in multi-step exploration for efficiently exploring the environment, while reducing the reconstruction error of the spatial process.
- It imposes visitation of intermediate goals as a routing problem for minimizing the traversed distance between two multi-exploration steps.
- It combines the strategy with efficient modelling using the GRBCM [29] to maintain online computational capabilities when exploring larger areas.

The remainder of the paper is organized as follows. Representation of a spatial process as GP is discussed in Section 2. A detailed description of the introduced system and its functionalities are given in Section 3, along with the exploration objectives. Evaluation setup and results followed by discussion are presented in Section 4, Section 5 and Section 6. Conclusion of the paper is given in Section 7, where the proposed method and its possibilities for further development are discussed.

## 2. Gaussian Process Regression

### 2.1. Gaussian Process

A GP is any finite collection of random variables that has a joint multivariate Gaussian distribution [9]. It is a very popular and well-known non-parametric statistical method to model spatial dependencies of a physical process. It is a distribution over functions [30] fully characterized by its mean function m(x) and covariance function $k(x,x^{\prime },\theta )$ for any pair x, $x^{\prime }$ representing spatial locations and some hyperparameters θ [9].

We assume that the value of m(x) is set to zero, which implies a priori no known values of the observed process. In this work, we are interested in modelling smooth processes. Accordingly, we employ the covariance function as a SE [9]:

(1) $$k\left(x,x^{\prime },\theta \right)=\sigma_{f}^{2}\exp\left(-\frac{||x-x^{\prime }{\left|\right|}^{2}}{2l^{2}}\right)+\sigma_{n}^{2}\delta_{xx^{\prime }},$$

where $\delta_{xx^{\prime }}$ is Kronecker’s delta, and $\theta ={\left[\sigma_{f}^{2},l,\sigma_{n}^{2}\right]}^{T}$ represents hyperparameters. For different covariance functions (Matérn, Rational Quadratic, etc.) and their characteristics, we refer the reader to [9].

SE hyperparameters are defined as follows: (i) The signal standard deviation $\sigma_{f}^{2}$ dictates the variation of function values from the mean; (ii) the characteristic length-scale *l* represents how smooth the function is and informally “how close” two points x and $x^{\prime }$ have to be for influencing each other significantly; (iii) variance of the sensor noise fluctuations $\sigma_{n}^{2}$ represents expected level of noise present in the data [9].

Next, we present the following definitions: $X={\left[x^{\left[1\right]},x^{\left[2\right]},\cdots ,x^{\left[N\right]}\right]}^{T}$ is a matrix where each row corresponds to a spatial location where measurement data was acquired by the robot, $z={\left[z^{\left[1\right]},z^{\left[2\right]},\cdots ,z^{\left[N\right]}\right]}^{T}$ are the corresponding *N* measurements, and $X_{*}={\left[x_{*}^{\left[1\right]},x_{*}^{\left[2\right]},\cdots ,x_{*}^{\left[R\right]}\right]}^{T}$ is a matrix where rows correspond to *R* “test” locations—spatial points where we want to predict process values using the learned model.

Now we use $k(x,x^{\prime },\theta )$ to define matrices that evaluate cross-correlations: (i) K—between measurement points X; (ii) $K_{*}$ —between measurements X and “test” points $X_{*}$; (iii) $K_{**}$ —between “test” points $X_{*}$. As a remark, matrices K, $K_{*}$, and $K_{**}$ are all implicit functions of θ due to their dependency on the covariance function $k(x,x^{\prime },\theta )$.

Our aim is to predict process values $y_{*}$ and the corresponding uncertainties at “test” locations $X_{*}$, given obtained measurements z and associated locations X. Before being able to predict the process with the GP model, we need to learn it. This implies utilizing training data, X and z, to determine the hyperparameters. Given the training data, we can estimate the suitable hyperparameters $\theta_{*}$ for our measurements. The learning phase is also known as model training. The hyperparameter estimation is typically executed via gradient-based optimization aimed at maximizing the log-marginal likelihood function of θ [9]:

(2) $$\theta_{*}=\underset{\theta }{\mathrm{argmax}}\left\{-\frac{1}{2}z^{T}K^{-1}z-\frac{1}{2}\log\left|K\right|\right\}.$$

Finally, the prediction $y_{*}$ is based on $\theta_{*}$ (Equation (2)). The vector $y_{*}$ is a random vector with the following probability density function: $p(y_{*}|X_{*},X,z)=N$ $\left(\mu_{*},\Sigma_{*}\right)$, where $\mu_{*}$, $\Sigma_{*}$ are calculated as (more details in  [9]):

(3) $$\begin{array}{cc} \mu_{*} & =m\left(X_{*}\right)+K_{*}^{T}K^{-1}\left(z-m\left(X\right)\right),\\ \Sigma_{*} & =K_{**}-K_{*}^{T}K^{-1}K_{*}. \end{array}$$

The main drawback of using a GP in real-world applications is its poor scalability with respect to data size. Given *N*, its complexity during the training phase is $O(N^{3})$ as we need to invert the N×N dimensional covariance matrix. Moreover, due to matrix-vector operation, complexity of its prediction phase is $O(N^{2})$.

### 2.2. Large-Scale Gaussian Process Regression

In order to apply a GP without limiting the size of the operated environment, we apply GRBCM [29], an aggregation method that allows us to combine independently trained GP regressors from multiple data sets.

Firstly, given an initial dataset D of size *N*, it is divided into *P* subsets, each with size $p_{0}$: One communication disjoint partition $D_{c}=D_{1}$ generated as a random subset, and disjoint partitions $D_{p}=\left\{X^{\left(p\right)},z^{\left(p\right)}\right\},p\;\geq 2\ldots P$ generated by k-means clustering [31]. Secondly, the data-sets ${\left\{D_{p}\right\}}_{p}^{P}$, p≥2 in GRBCM are extended with a communication dataset as enhanced sets $D_{+p}=\left\{D_{c},D_{p}\right\}$.

The GP regression (Equation (3)) is then applied on communication partition $D_{c}$, producing a global communication expert $E_{c}$ trained on $D_{c}$ that captures the main features of the target function. Furthermore, it is applied to each disjoint partition $D_{+p}$, leading to the second expert type—local experts. Those experts ${\left\{E_{p}\right\}}_{p=2}^{P}$ locally refine predictions trained on respective $D_{+p}$.

It is important to mention the assumption that local experts $E_{p}$ are uncorrelated, leading to the following predictive distribution equations for aggregated model [29]:

(4) $$\begin{array}{cc} \mu_{*,A}\left(x_{*}\right) & =\sigma_{*,A}\left(x_{*}\right)\left\{\sum_{p=2}^{P}\beta_{p}\sigma_{+p}^{-2}\left(x_{*}\right)\mu_{+p}\left(x_{*}\right)-\left(\sum_{p=2}^{P}\beta_{p}-1\right)\sigma_{c}^{-2}\left(x_{*}\right)\mu_{c}\left(x_{*}\right)\right\},\\ \sigma_{*,A}^{-1}\left(x_{*}\right) & =\sum_{p=2}^{P}\sigma_{+p}^{-2}\left(x_{*}\right)-\left(\sum_{p=2}^{P}\beta_{p}-1\right)\sigma_{c}^{-2}\left(x_{*}\right), \end{array}$$

where $\beta_{p}$ is defined as [29]:

(5) $$\beta_{p}=\left\{\begin{array}{cc} 1, & p=2\\ 0.5(\log\;\sigma_{c}^{2}\left(x_{*}\right)-\log\;\sigma_{+p}^{2}\left(x_{*}\right)), & 3\leq p\leq P. \end{array}\right.$$

In summary, the global communication expert captures the main features of the model, while the local experts are improving local predictions. Generally, for aggregation models, calculating local experts principally has the same complexity as calculating a single global expert—cubic in model training and quadratic in prediction. However, a significant scalability improvement lies behind two reasons: (i) The size of local experts is much smaller than the full dataset N ($p_{0}\ll N$), and (ii) local experts can be computed in parallel and in a distributed manner. For more details about GRBCM algorithm refer to [29].

## 3. Integrated Exploration

In this section we describe our proposed algorithm for autonomous exploration of a spatial process in an unknown environment. First, we present an overview of the system. Second, we describe the elements that compose our strategy in detail.

We aim to explore a spatial physical process y at *R* corresponding locations $X_{*}\subset R^{2\times R}$ where we want to obtain process values which can be modelled as a GP (Section 2.1). For large-scale spatial physical processes we construct the GP as an aggregated modelling of individual experts with GRBCM (Section 2.2).

Additionally, we assume that the structure of the operating environment is a priori unknown. That is, information about the position of obstacles is unavailable. The lack of information about the environment motivates us to extend process exploration with mapping. Note that an accurate map M is crucial for exploration planning and safe navigation through the environment. Furthermore, a robot must localize itself with respect to such a map by means of on-board sensors as an external localization system is unavailable. We would like to remark here that we assume the ability of the on-board localization system to maintain the position uncertainty below the process correlation length. This assumption permits us to focus on our aforementioned strategy. Meaning, that for considered environments, enhanced odometry is sufficient for localization. Nevertheless, we will study localization component as a part of our future work.

We consider a ground-based mobile robotic platform equipped with an on-board computer capable of real-time computations. It carries a 3D LIDAR that provides point clouds in the local sensor frame, and a magnetic field sensor that collects one process measurement $z^{\left[n\right]}$ (observed value of the process y) per location $x^{\left[n\right]},n\;=1,\ldots ,N$.

**Figure 1:**
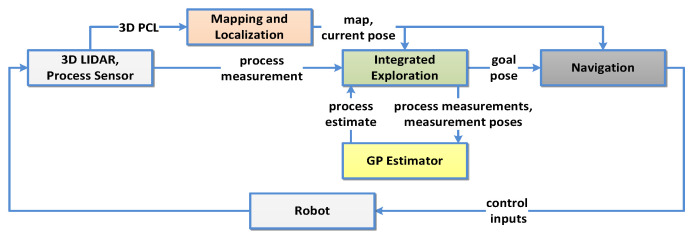
System overview.

Figure 1 shows an overview of the complete system. Inputs from on-board sensors are directly forwarded to the module for environment mapping (Mapping and Localization) and indirectly to the process estimation module (GP Estimator) through the exploration module (Integrated Exploration). Details of these two modules are explained in Section 3.2. Their output is used by the key module of our system—the Integrated Exploration module.

The Integrated Exploration module has the following tasks: (i) Point of Interest (POI generation $X_{\mathrm{poi}}=\left\{x^{\left[j\right]},\;j\;=1,\ldots ,G\right\}$, where *G* is the number of POIs; and (ii) sort $X_{\mathrm{poi}}$ in a specific way to obtain an ordered sequence of POIs as goal poses $X_{\mathrm{goal}}=\left\{x^{\left[g\right]},g\;=1,\ldots ,G\right\}$. More details about the exploration module are provided in Section 3.4. Further, goal poses are forwarded sequentially to the Navigation module responsible for local path planning and execution by providing control inputs for autonomous movement of the robotic platform.

The robot starts its exploration of the environment at a certain known initial position $x^{\left[0\right]}$, obtaining initial scan cloud $P^{\left[0\right]}$ and process measurement $z^{\left[0\right]}$. During exploration, it continuously observes the environment through its perception sensor and localizes itself within the area. In parallel, the robot takes measurements of the process $z^{\left[g\right]}$ at locations $x^{\left[g\right]}\in X_{\mathrm{goal}}$. This loop continues until a stopping criterion is fulfilled. For details of the algorithm description refer to Section 3.4.

### 3.1. Sensors and Robot

#### 3.1.1. Sensors

We employ two sensors: (i) Perception sensor—responsible for mapping the environment and obstacle avoidance, and (ii) process sensor—responsible for collecting spatial process information (i.e., magnetic field measurements). For the former, we have a 3D LIDAR sensor with 360° horizontal, and 30° vertical FOV with a range up to 100 m. However, any other perception sensor that provides distance from obstacles may be applied. For the latter, we consider a magnetic field sensor that collects information point-wise, i.e., only at the current location.

#### 3.1.2. Robot

**Figure 2:**
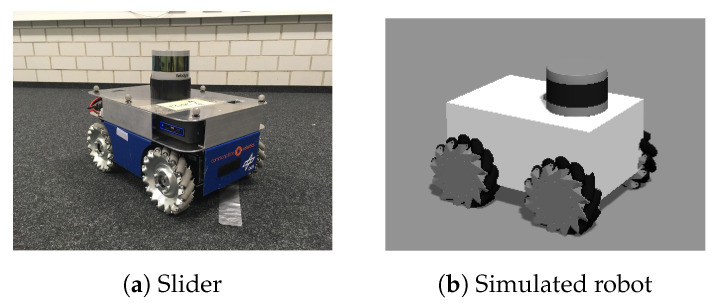
Robotic platform equipped with Velodyne VLP-16 and an on-board computer.

We use a holonomic robotic platform (Figure 2) equipped with sensors and an on-board computer for autonomous localization, mapping, navigation and exploration execution (Section 3.2, Section 3.3 and Section 3.4). Generally, any robotic system that explores in 2D can be applied if equipped with aforementioned required components.

### 3.2. Mapping and Localization, Navigation

#### 3.2.1. Mapping

For navigation and exploration purposes, we employ an occupancy grid representation of the map M. An occupancy grid map is a two-dimensional grid representation of the environment, holding a probabilistic information of each cell occupancy, which can be marked as: (i) Free, (ii) occupied, or (iii) no information [32].

To construct an occupancy grid, we use point cloud inputs $P^{\left[1:t\right]}$, where *t* is current input time from the 3D LIDAR, which we then vertically project onto 2D xy horizontal plane given robot’s height dimension constraints. Note that before projecting input clouds $P^{\left[1:t\right]}$, we apply ground base filtering (by extraction of ground points) and height filtering (by extracting points above certain height). This projection is used for safe navigation through the environment with obstacles of various shape and size.

#### 3.2.2. Localization

Pose $x^{\left[t\right]}$ at time *t* is represented as a 2D position [x,y] in Cartesian coordinate system, and yaw as orientation. Odometry and translation/rotation estimation of the incoming point cloud $P^{\left[t\right]}$ is carried out with respect to the reference cloud $P^{\left[t-s,t-1\right]}$ (cloud of processed, i.e.,  filtered and concatenated, *s* previously registered scans). For this, a registration algorithm—the point-to-plane variant of ICP algorithm [33]—is employed. Note that during exploration, relative localization with respect to initial pose $x_{0}$ is performed. One can choose from a selection of readily available state-of-the-art vision and LIDAR-based localization/SLAM algorithms such as LOAM [34], Lego-LOAM [35], SegMap [36], or Cartographer [37] for maintaining localization accuracy in GPS-denied environments.

#### 3.2.3. Navigation

We employ a combination of optimal global A* planner [38] on the grid with TEB reactive local planner [39]. Waypoints are fed to the Navigation module by the Integrated Exploration module (Section 3.4). Additionally, it receives periodical updates from Mapping and Localization module (previously described), assuring no-collision paths.

### 3.3. GP Estimator

At any time *t*, from the set of collected measurements $z^{\left[1:t\right]}={\left[z^{\left[1\right]},\ldots ,z^{\left[t\right]}\right]}^{T}$ and its estimated positions $X^{\left[1:t\right]}={\left[x^{\left[1\right]},\ldots ,x^{\left[t\right]}\right]}^{T}$, we can predict the process values $y_{*}$ and corresponding uncertainties at probe positions $X_{*}$ (Equation (3)). Furthermore, in our case, hyperparameters are known and learned a priori. Let us remark that hyperparameters could be also learned online from measurements (Equation (2)).

As presented in Section 2.2, in order to cope with scalability, we extend our system with GRBCM [29]—a state-of-the-art aggregative method for the GP that synthesizes predictions from individually trained experts, where experts are trained on sub-sets of the full data set. Predictive distribution for GRBCM is estimated according to Equation (4).

### 3.4. Exploration Strategy

The aim of our integrated strategy is to address the problem of reconstructing a spatial GP in an a priori unknown environment while simultaneously exploring it. We assess the behaviour of the integrated strategy with respect to the two main objectives and its performance towards minimizing the total travelled distance while satisfying both:

1. Efficient spatial process exploration: Minimization of the process error in comparison to the ground truth.
2. Efficient coverage strategy: Increasing coverage of the environment map to reduce unknown portions of the map.

**Figure 3:**
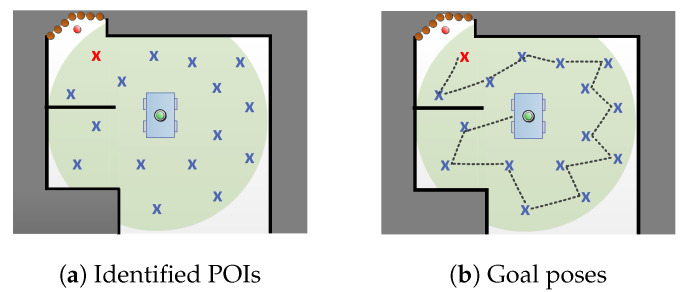
Main components of our exploration strategy. (**a**) Current position $x^{\left[t\right]}$ is marked with a green circle, light red cross marks goal location $x_{G}$ within planning radius *r* (shaded green area), blue crosses represent POI $X_{\mathrm{poi}}$, while orange circles are frontier with its centroid $x_{f}$ (red circle); (**b**) optimized by distance travelled, $X_{\mathrm{poi}}$ are represented as ordered points, i.e., goal poses $X_{\mathrm{goal}}$, which describe the sequence that the robot follows, obtaining measurement $z^{\left[g\right]}$ on each $x^{\left[g\right]}$.

As already mentioned, this integrated strategy consists of two key steps regarding POIs: (i) Identification (Section 3.4.1) and (ii) ordering—determining goals (Section 3.4.2). Results of both steps are visualized in Figure 3.

#### 3.4.1. POI Detection

In order to tackle our joined exploration objective, we select POIs in which taking measurements will improve estimate of the (i) current map (by considering frontiers of the map) and (ii) process (by selecting informative candidates on the estimated spatial field):

**Frontier.** First, to efficiently map the environment, we select classical frontiers, which maximize map coverage [6]. For efficient detection of frontiers, which separate known regions from unknown in M, we apply WFD, a graph-based approach based on BFS [40]. This algorithm performs a search only on cells that are not yet traversed and represent free space, thus avoiding an expensive full map search. We weigh and sort frontiers by distance from the current location of the robot $x^{\left[t\right]}$ to their centroids—the average position of all frontier points for a given frontier—and choosing the closest one. The distance is calculated with the A* path-planning algorithm.

The shortest path to the centroid $x_{f}$ of the selected frontier provides a fast way to increase map coverage, but it drastically limits the area from which measurements of the process are obtainable. Since we can only collect process measurements point-wise given the sensor footprint, following the shortest way towards the frontier will not be the most informative path regarding process exploration. A lot of areas along the way will consequently be missed. Therefore, a trade-off between the shortest and the most informative path is considered by expanding the path from $x^{\left[t\right]}$ towards $x_{f}$ with a set of possible positions where the robot may obtain new informative process measurements while advancing the map coverage.

**Candidates (POI).** Searching for all possible process measurement locations X on M is infeasible for large maps. Moreover, we need to keep in mind that map and process estimations are changing with robot traversing forward, meaning that planning over a too long horizon may produce an inefficient exploration strategy due to significant changes in the map.

To overcome this, we limit the exploration of the process to a finite horizon *r* with respect to the robot position $x^{\left[t\right]}$ at any given time *t*. To identify potential POI candidates with a high information value, we apply BFS for traversing only through obstacle-free explored cells in M within the horizon *r*. We sample $X_{\mathrm{poi}}\subseteq X_{\mathrm{free},r}\subseteq$ $X_{r}\subseteq X$, where $X_{r}$ represents all measurement locations within the radius, while $X_{\mathrm{free},r}$ represents its subset of reachable locations (Algorithm 1, below). Here, $X_{\mathrm{poi}}$ is the set of selected measurement locations where all the surrounding cells within the inflation radius are unoccupied. If the frontier centroid $x_{f}$ is within radius *r*, it will be included in $X_{\mathrm{poi}}$.

As a metric for informativeness of each candidate, we define the information value of a position $x_{*}$ as the Shannon entropy of the posterior of the GP at that position:

(6) $$H\left(x_{*}\right)=\frac{1}{2}\ln\left(2\pi e\sigma_{x_{*}}^{2}\right),$$

where $\sigma_{x_{*}}^{2}$ represents the variance of the estimated process $y_{*}$ at location $x_{*}$. A high value of entropy here represents a high uncertainty of the process (i.e., non-observed cell). Variance $\sigma_{x_{*}}^{2}$ is estimated with GP regression (Section 2.1). Here, we emphasize that due to previously mentioned scalabilty limitations of classical GP regression approach, for a larger number of measurements we apply GRBCM that allows us to keep real-time performance of the algorithm.
**Algorithm 1**$X_{\mathrm{poi}}$ informative candidates sampling.**Require:** current location $x^{\left[t\right]}$, map M, radius *r*, sampling distance *k*, threshold ϵ **Ensure:** POI $X_{\mathrm{poi}}$  1:  Extract $X_{\mathrm{free}}$ from the M.  2:  $X_{\mathrm{poi}}=\left\{x^{\left[t\right]}\right\}$  3:  BFS ($X_{\mathrm{free}},x^{\left[t\right]}$):  4:       $\hat{x}=\left\{x_{*}\in X_{\mathrm{free}}:H\left(x_{*}\right)>\epsilon ,∥x_{*}-x^{\left[p\right]}∥\geq k,\forall x^{\left[p\right]}\in X_{\mathrm{poi}},\right\}$  5:       $X_{\mathrm{poi}}=X_{\mathrm{poi}}\cup \left\{\hat{x}\right\}$


We sample with a minimum separation distance *k* with respect to points $x^{\left[p\right]}$ already in $X_{\mathrm{poi}}$, where *k* is chosen depending on the hyperparameters θ, particularly by *l*, a length-scale that defines a process correlation, informally representing “how far we can reliably extrapolate from obtained data” (Section 2.1). Values below threshold ϵ (also dependant on hyperparametrs) represent low entropy, i.e., low process uncertainty.

#### 3.4.2. Goals Detection

Finally, once the POIs are selected, the robot needs to visit them all while minimizing the distance. To achieve this, POIs first need to be ordered as further described in this section. Ordered POIs are represented as goals $X_{\mathrm{goal}}$.

**Distance matrix.** In order to find the optimal solution to traversing all $X_{\mathrm{poi}}$ in terms of the distance travelled, we calculate the distance matrix between each pair of POIs $x^{\left[i\right]},x^{\left[j\right]}\in X_{\mathrm{poi}}$, including the current pose $x^{\left[t\right]}$. The distance between $x^{\left[i\right]}$ and $x^{\left[j\right]}$ is calculated with A* algorithm, where i,j∈[1,…,G].

**Route calculation.** Once the matrix is created, we formulate our problem as a routing problem. Since TSP does not consider distinguishable depots for both start and finish, we apply a simplified case of VRP [41]—a generalized version of TSP problem. More specifically, we apply a variant of the multi-depot VRP introducing additional constraints on start/end depots to minimize travelling distance from current location $x^{\left[t\right]}$ to goal location $x_{G}$ while visiting all $X_{\mathrm{poi}}$. We call it “vanilla” VRP with only one vehicle with given constraints to minimize the distance travelled between start and finishing depots.

The reason for imposing additional constraints on the depots (start and finish location) for the solver is to indicate that we are not interesting in returning to the starting location, but rather to a specific location aligned with our exploration objectives. Depots are determined as follows:

- As a start depot, set the current location $x^{\left[t\right]}$.
- If the frontier centroid $x_{f}\in X_{\mathrm{poi}}$, then $x_{G}=x_{f}$.
- Otherwise, we set $x_{G}$ to be $x^{\left[p\right]}\in X_{\mathrm{poi}}$, that has the shortest distance to frontier centroid $x_{f}$ (to preserve the direction favoring area coverage).

**Figure 4:**
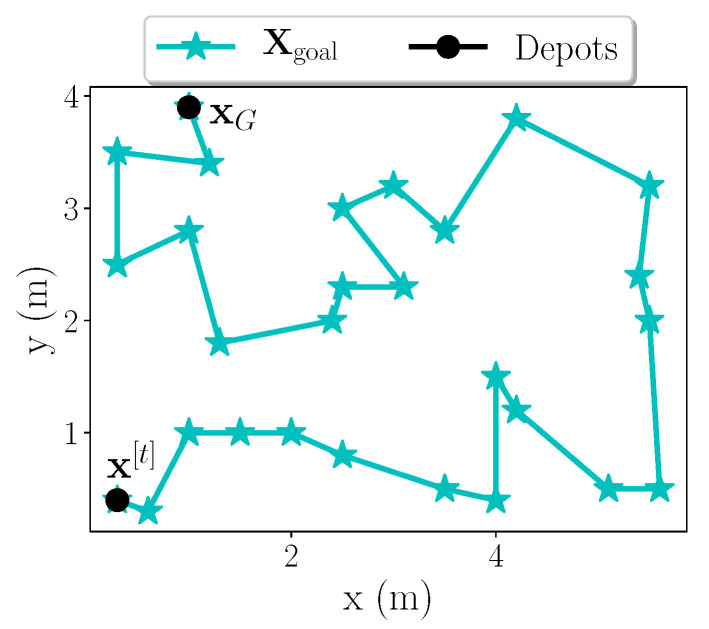
One-robot solving a multi-depot “vanilla” VRP: $x^{\left[t\right]}$—current location; $x_{G}$—goal location. Goals ($X_{\mathrm{goal}}$) are represented with blue asterisks and depots with black circles.

The output of “vanilla” VRP solver is a vector $X_{\mathrm{goal}}$ of sorted waypoints. Figure 4 depicts a simple example of the solver solution where constraints are only applied on the exact starting and ending depot for a one-robot system.

### 3.5. All Components of Our Integrated Exploration Strategy

**Figure 5:**
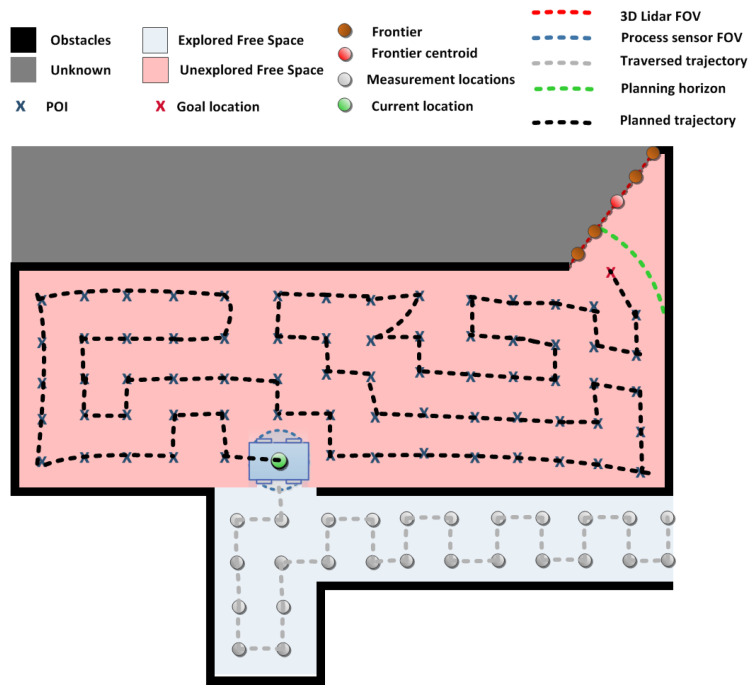
All components of a single multi-step of our integrated exploration strategy: Grey circles represent previously obtained measurements and the area around those circles is considered explored (light blue). Robot, at the current location (green circle), samples Point of Interest (POI, blue crosses)—in the obstacle-free region of the map, where the process is unexplored (red area)—within radius *r* (green line). The routing algorithm provides a multi-step exploration solution (black dotted line) from the current location to the goal location (red cross). The goal location is the POI closest to the frontier (brown circles) and the corresponding centroid (red circle). The frontier is defined by the LIDAR FOV (red dotted line). Obstacles are marked by a black line and unexplored area in grey.

Let us consider an example scenario where the robot has already partially explored the environment (Figure 5). The green circle represents the robot during the experiment, with current time *t*. The grey circles, along with the green one, represent visited locations $X^{\left[1:t\right]}=\left[x^{\left[1\right]},\ldots ,x^{\left[t\right]}\right]$ with their observed process value measurements $z^{\left[1:t\right]}=\left[z^{\left[1\right]},\ldots ,z^{\left[t\right]}\right]$. Now, we also have an environment map M with regions marked as free, occupied or unknown. Given our planning horizon (radius *r*), all $X_{\mathrm{poi}}$ are detected (Section 3.4.1). This corresponds to the exploration objective of obtaining measurements at the most informative points. However, we still need to answer the question: “What is the most efficient order to visit them with objectives of minimizing the travelled distance and increasing the map coverage?” This is where the knowledge about frontiers comes in place. We can exploit the frontier in order to guide robot towards the goal position that is closest to the “border between the known and unknown” which guarantees an increase in map coverage (Section 3.4.2).

However, we need to deviate from the shortest path in order to collect process samples. Therefore, the start depot $x^{\left[t\right]}$ and end depot $x_{G}$ (guiding towards $x_{f}$) are used as inputs to “vanilla” VRP. Note that if we would neglect knowledge about $x_{f}$, the robot could finish its exploration e.g., in the far left bottom corner (Figure 5), which means it would need to traverse a significant distance towards unknown regions in order to proceed with exploration.

#### Algorithm Work-Flow

**Figure 6:**
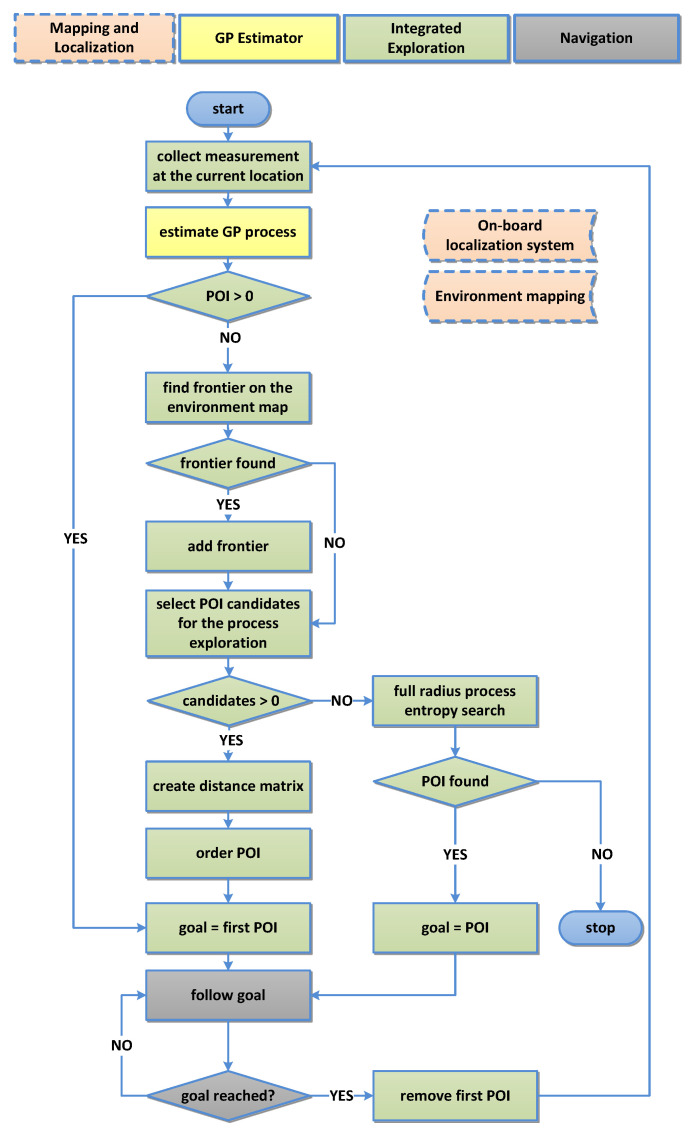
Algorithm state diagram. Colored blocks represent the following: Red—mapping and localization; yellow—estimation of GP; green—core algorithm (our integrated exploration); and grey—navigation.

Figure 6 describes the algorithm that controls the behaviour of the robot. We can summarize it as follows:

- Mapping and Localization: The robot continuously perceives the environment and accordingly updates the map M and its current location estimate $x^{\left[t\right]}$.
- Navigation: Until any unvisited $x^{\left[g\right]}\in X_{\mathrm{goal}}$ exists, it continues following precomputed goal poses $X_{\mathrm{goal}}$ (ordered representation of $X_{\mathrm{poi}}$).
- At each $x^{\left[g\right]}$ reached, collect the process measurement $z^{\left[g\right]}$.
- GP Estimator: Estimate GP process at probe locations $X_{*}$ over the whole environment.
- Integrated Exploration If $X_{\mathrm{goal}}$ is empty, detect the next frontier on M according to the procedure described in Section 3.4.1 and:
  − Sample locations within *r* as described in Section 3.4.1—producing unordered $X_{\mathrm{poi}}$, a list of candidates where we want to obtain our next measurements to increase knowledge about the process.
  − From $X_{\mathrm{poi}}$, create a distance matrix, representing computed distances between POI.
  − Order POI according to the procedure described in Section 3.4.2 so that all POI are visited and total travelled distance is minimized, resulting in $X_{\mathrm{goal}}$.
  − If Algorithm 1 finds no suitable candidates within the limited horizon *r*, extend the horizon to cover all discovered cells $X_{free}$ on the map. Select only the closest candidate location that satisfies H(x)>ϵ as the next goal location $x^{\left[g\right]}$. Otherwise terminate the mission.

## 4. System Evaluation

We first evaluate the proposed strategy in various simulation scenarios. Then we carry out an experiment, in which a mobile holonomic robot operates in an unknown environment populated by obstacles to explore magnetic field intensity. As a remark, during evaluation our proposed strategy will be referred to as Integrated Exploration (IE).

We assess the following:

1. What is the scalability of GRBCM for exploration of spatial processes?—Simulations (Section 5.1).
2. What is the correlation between sampling distance *k* and error decrease in the process reconstruction for the IE strategy? How does it affect total exploration distance?—Simulations, experiment (Section 5.2 and Section 6.2).
3. How does the IE perform against the benchmarks in various scenarios?—Simulations, experiment (Section 5.3.3 and Section 6.2).

The scenarios considered in the evaluation are listed in Table 1.

### 4.1. General System Setup

#### 4.1.1. Robotic Platform

For experiments we use a holonomic ground robotic platform equipped with an Intel Nuc (Intel Nuc https://www.intel.de/) (Figure 2a), further referred to as a “slider”. A simulated representation of the robot with mechanum wheels is made in Gazebo (Gazebo http://gazebosim.org/), based on the characteristics of the real-robotic platform (Figure 2b).

#### 4.1.2. Perception Sensor

Mapping and localization is carried out with the 3D LIDAR Velodyne VLP-16 (Velodyne https://velodynelidar.com/vlp-16.html).

#### 4.1.3. Process Sensor

Resolution of the simulated process sensor is 0.1 m and measurements $z^{\left[g\right]}$ per location $x^{\left[g\right]}$ are obtained for various sets of predefined ground truth data which represent the GP (Table 2). The ground truth positions are estimated by Gazebo and the motion tracking system VICON (VICON https://www.vicon.com/) for simulations and experiments, respectively. Moreover, regarding Table 2, Process 1 and Process 2 measurements are samplings from a simulated multi-variant GP and the magnetic field data corresponds to a previously collected real-world magnetic field intensity [8].

Software components of the system are implemented using ROS (ROS—Robot Operating System. http://wiki.ros.org/.), Gazebo, C++, and Python 2.7. The process estimator is implemented by employing an open-source GP framework GPy [42]. Lastly, in order to solve the routing problem, we utilize the OR-Tools [43] (library developed and maintained by Google).

## 5. Simulations

### 5.1. Scalability of Gaussian Processes for Spatial Modelling

The aim of our first simulation tests is to validate the performance of GRBCM in predicting spatial processes in comparison with a full GP prediction.

In order to evaluate the GRBCM performance on a large data set, we consider a simulated 2D multivariate GP dataset with N=7592 measurement points and R=214500 (390×550) “test” locations. As a reminder, for the GRBCM we have P−1 local experts $E_{p}$ and one communication global expert $E_{c}$. We assume the same size for all experts, i.e., for a dataset size *N*, the size of each is $p_{0}=N/P$.

Simulation tests are performed on Dell XPS 13 7390 laptop with Linux operating system Ubuntu 18.04LTS, 10th Generation Intel(R) Core(TM) i7-10510U, and 16 GB RAM.

#### Simulation Results

We present results in Table 3 that show a significant improvement in the time required to estimate the process, with only a slight increase in the final NMSE of the reconstructed process. Moreover, it is observed that the NMSE error decreases when assigning more observations to each expert, i.e., increasing $p_{o}$. However, we note that more observations per expert lead to increased computation costs due to matrix inversion. Furthermore, we see that even with a smaller $p_{o}$, time does not necessarily decrease. This is due to the increased total number of experts *P* and multiple parallel computing processes. Therefore, by finding a good trade-off between the minimum size of experts and computational capabilities, we can obtain an accurate estimate for large-scale processes with prominently low computation and time costs.

In our further simulations, process estimation with GRBCM is carried out with a fixed expert size $p_{0}$ of 350. For N<350, we have only one expert, which gives an equivalent performance to classical GP.

### 5.2. Sampling Distance

Since our aim is to consider real-world scenarios, we only want to collect important information. We ask ourselves what is the minimal distance between samples *k* that will lead to an accurate reconstruction of the process but at the same time balance the number of samples we need to obtain. We vary *k* with respect to the length-scale *l*. In such a scenario, we obtain observations about Process 1 (S1: Table 1) with l=0.2 m, applying our IE strategy in an empty room with a full horizon. For all runs, the starting position is (x(m),y(m))=(−3.3,−3.3).

#### Simulation Results

**Figure 7:**
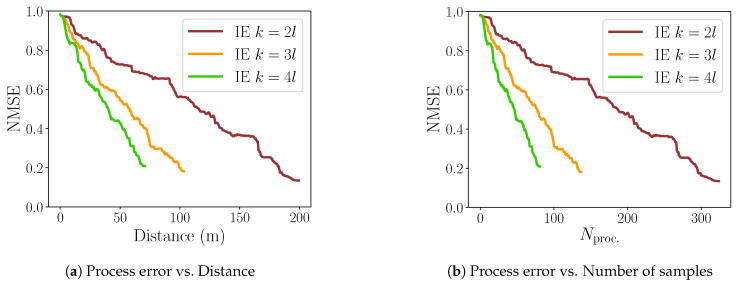
Variation of sampling distance parameter *k*.

Figure 7 presents the results with a varying sampling step k=[2l,3l,4l]. With IE, the number of samples $N_{\mathrm{proc}}.=\left[325,138,82\right]$ are obtained and the corresponding NMSE in relation to the ground truth are [0.13, 0.18, 0.21]. The travelled distances are [199.45 m, 103.57 m, 70.82 m]. We can notice that the higher the sampling distance *k* is, the steeper the NMSE curve is with respect to the distance (Figure 7a) and the number of samples *N* (Figure 7b). At the same time, the final attained NMSE is larger with larger distances. Let us remark that NMSE will never reach zero due to physical limitations of the robot, which is constrained not to collect data closer than 0.5 m to the obstacles in the environment.

For the rest of the experiments we select k=3l, which gives us an optimal trade-off between final process accuracy and distance travelled.

### 5.3. Evaluation of the Strategy in Simulation

#### 5.3.1. Applied Baselines

In the literature for GP exploration, entropy-based methods [10,17,44] are typically used and they are showing dominance over random methods. Moreover, the baseline for map exploration originates from the frontier-based method [6], which is used in many map exploration (i.e., [17,20]) and active SLAM approaches (i.e., [5,15]).

Thus, in order to validate the performance of our integrated exploration strategy, we consider a combination of entropy driven and frontier-based baseline strategies as follows:

**GGE**. At each step, selecting one $x_{g}$ from all possible reachable cells $X_{free,r}$ within radius covering all discovered cells r=full:

(7) $$X_{c}=\left\{x_{g}:x_{g}=\underset{x\in X_{c}}{\arg\;\max\;}H\left(x\right),\;s.t.H\left(x\right)>\epsilon \right\},\;$$

with stopping criteria $X_{c}=\left\{\right\}$, i.e., when there are no more cells satisfying Equation (7). This baseline is inspired by the maximum informativeness criterion [44] that guides the robot towards locations with the highest information gain while considering the global information available about the process.

**GLGE**. At each step, selecting one $x_{g}$ from all possible reachable cells $X_{free,r}$ within radius *r* covering all discovered cells that is the solution of:

(8) $$x_{g}=\underset{x\in X_{free,r}}{\arg\;\max\;}H\left(x\right),\;s.t.H\left(x\right)>\epsilon ,\;$$

with stopping criteria $x_{g}=0$, i.e., when there are no more cells satisfying Equation (8). This baseline is likewise inspired by the maximum informativeness criterion [44] yet with a small twist that guides the robot towards locations with the information gain above a priori set threshold, while considering the global information available about the process only if it cannot find a local suitable candidate.

**SS**. Goal pose is $x_{g}=x_{f}$, the centroid of the frontier. If at least one reachable frontier exists, measurements are collected each *k* meters distance travelled towards $x_{g}$, where parameter *k* is the sampling distance described in Section 3.4.1. Baseline for the map exploration frontier originates from commonly used frontier-based exploration, both in the map coverage tasks [6,17,20], as well as a part of active SLAM exploration component [5,15]. Once no more frontiers are presented, i.e., map is fully explored, the GLGE strategy is used to complete an entropy driven process exploration.

We would like to remark that the direct comparison with active SLAM algorithms that consider the localization uncertainty, regarding SS baseline, is not considered due to our assumption that the on-board localization system is able to localize the robot with satisfying accuracy, and therefore we neglect loop-closures and the need to revisit previously explored areas. A summary of the evaluated benchmarks along with IE strategy is presented in Table 4.

#### 5.3.2. System Simulation Setup

To examine the behaviour of each benchmark against our strategy on a smaller scale, we consider a simulated physical process (Process 1: Table 2) with a priori given hyperparameters. Simulations are carried out in two $8\times 8\;m^{2}$ environments with distinct degrees of obstacle complexity: (i) Obstacle-free (S1: Table 1) and (ii) room-like (S2, S3: Table 1). For these environments, 5 starting positions are chosen: (x(m),y(m))=(−3.3,1.0),(−3.3,−3.3),(0.0,0.0),(2.0,−2.3),and(2.5,−2.5).

Furthermore, to additionally evaluate our system on a larger scale, we test our IE strategy against GLGE in a large-scale environment (56.1×38.4 $m^{2}$) populated with obstacles, considering a simulated physical process (Process 2: Table 2) with a priori given hyperparameters.

The robot footprint, including a safety buffer, is set to radius of 0.5 m for all simulation scenarios.

Simulation tests S1, S2 (Table 1) are performed on computer with Linux operating system Ubuntu 16.04LTS, Intel(R) Core(TM) i7-3820, and 32GB RAM. Further simulation scenario S3 (Table 1) is performed on computer with Linux operating system Ubuntu 18.04LTS, Intel(R) Core(TM) i7-10510U, and 16GB RAM.

#### 5.3.3. System Simulation Results

**Obstacle-free small room-like environment** (S1 in Table 1). We assume that the process is fully explored when process NMSE reaches 0.18 with respect to the ground truth. We are not able to collect samples around borders due to physical limitations of the robotic platform.

As a remark, the SS benchmark is skipped for the obstacle-free scenario since there are no frontiers. Moreover, the map is explored already in the first step since perception sensor radius is larger than the environment size.

**S1 simulation results.** In Table 5 we observe that our IE strategy outperforms benchmarks with respect to the total travelled distance. While GGE collects the least amount of process samples $N_{\mathrm{proc}}.$ while reaching the same NMSE (making it computationally less expensive), it produces significantly longer trajectories. As a reminder, with GRBCM we can compensate computation costs introduced due to a higher number of process samples, thus making the total distance travelled our main comparison tool between different strategies.

**Figure 8:**
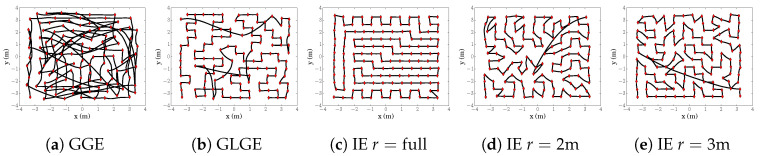
Example trajectory for scenario S1 and starting location (−3.3,−3.3) for the different strategies.

Looking into the example of the resultant trajectories (Figure 8) in the obstacle-free scenario (equivalent to exploring one big empty room), the behavior of GGE (Figure 8a) is stepping towards the highest value of H(x) while skipping some close locations with H(x)>ϵ, and forcing it in later stages to traverse longer distances. While GLGE (Figure 8b) strategy avoids initially traversing larger distances, it lacks a systematic approach and still misses multiple informative locations, making exploration more “expensive” in the final stages when the environment is fully observed, but the process itself is not yet fully explored in many previously visited local areas. If the room size is covered by search radius *r* in IE (Figure 8c), we can solve it efficiently within one multi-step exploration sequence, avoiding any expensive returns to the vicinity of previously explored locations. However, we can observe that even with a smaller radius we still systematically explore the horizon *r* before moving forward (Figure 8d,e), thus behaving likewise to GLGE only in cases when the local area is completely explored. Consequently, our strategy produces significantly shorter trajectories.

**Small room-like environment, obstacles introduced** (S2 in Table 1). In this evaluation, we assume that the process is explored when NMSE reaches 0.21 with respect to the ground truth. As before, we are not able to collect samples in close proximity of obstacles.

**S2 simulation results.** When we add obstacles, we observe that IE tends to produce lower process error for the same map coverage (Table 6). We also see that our strategy collects more samples and traverses larger distances before the map is fully explored. This is due to its focus on exploring the surrounding process before continuing further towards unexplored areas. The compared benchmarks favor moving faster towards regions with high process uncertainty while ignoring some less uncertain but still important areas around its current measurement location. As a consequence of exploring room by room before moving further, IE also produces shorter final trajectories (Table 6).

**Figure 9:**
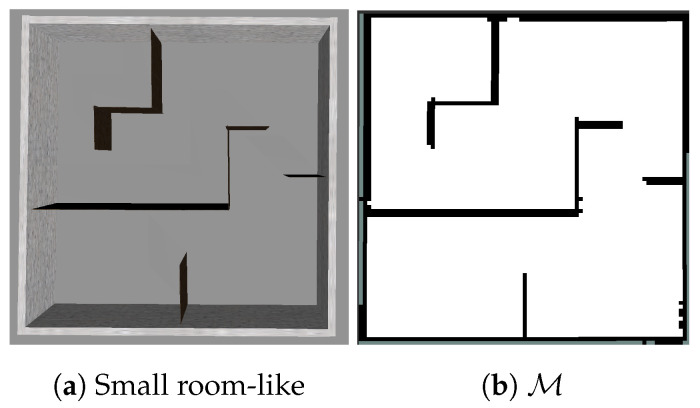
Environment for scenario S2.

**Figure 10:**
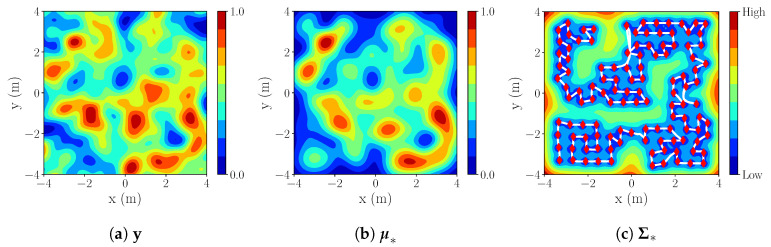
S2: IE r=3m,k=3l with red diamonds in (c) representing measurements and white lines trajectory.

An example of IE mapping for the starting position (x(m),y(m))=(−3.3,−3.3)) with r=3 m is shown below (Figure 9). For this setup, we see the results of the reconstructed process $y_{*}$ (based on Process 1—Figure 10a), represented by mean (Figure 10b) and variance (Figure 10c).

**Figure 11:**
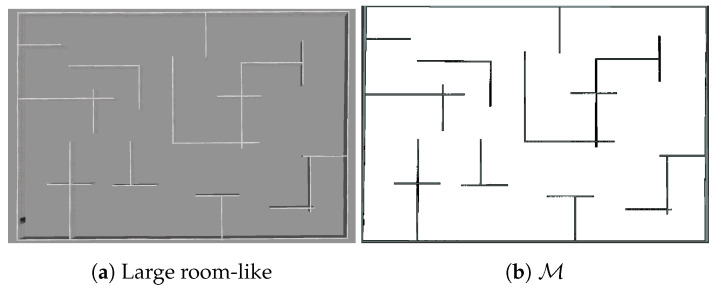
Environment for scenario S3.

**Large room-like environment, obstacles introduced** (S3 in Table 1). In addition to the multiple tests performed with S1 and S2, we test our strategy against GLGE in a large scale environment covering a surface of 2154.24 $m^{2}$ (Figure 11). We assume that the process is explored when process NMSE reaches 0.084 with respect to the ground truth.

**Figure 12:**
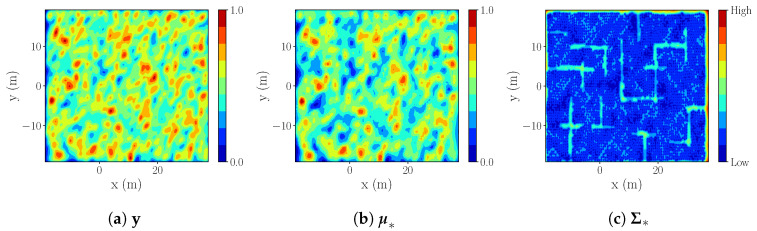
S3: IE r=3m,k=3l.

**S3 simulation results.** We note a lower final NMSE error in S3, in comparison with S1/S2. This is due to higher process correlation in process 2, resulting in better estimation of unreachable areas $\left\{X\backslash X_{\mathrm{free}}\right\}$. The estimated process for r=3 m. is shown in Figure 12.

Despite our IE strategy (r=[3,6] m) requiring more measurements to reach the same NMSE as GLGE (Table 7), GLGE produces more than a 100 m longer trajectory for exploring the full environment, making our strategy more efficient regarding distance measure.

**Figure 13:**
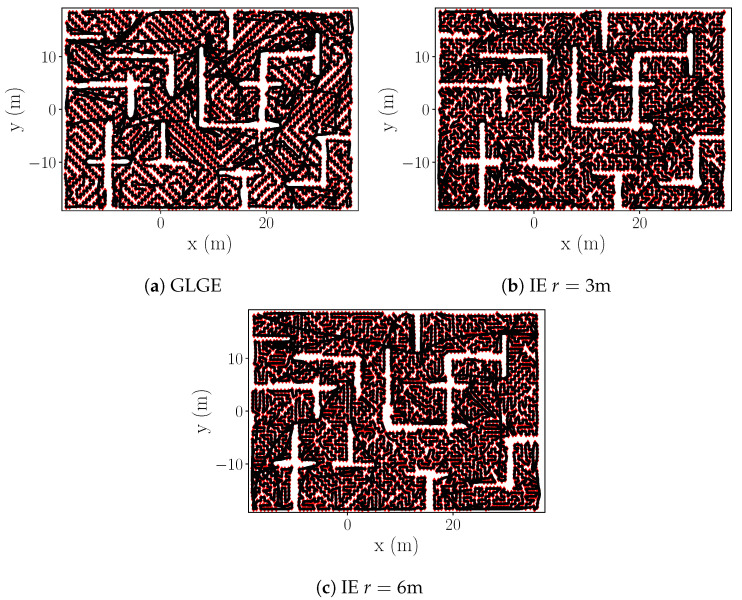
S3: Trajectories (black lines) and obtained measurements (red markers).

The reason for GLGE placing fewer measuring points lies in its greedy movement strategy, as it always places only one next best point to satisfy Equation (8). In contrast, our strategy places as many points as possible within the minimum sampling distance that are above the threshold ϵ (Algorithm 1). As a trade-off, more points are generated, but a smaller distance is needed to visit all points. The executed trajectories are shown in Figure 13.

As a final remark, we consider a different final NMSE for each scenario for stating the process as fully explored. Given the same minimum sampling distance *k*, there are two main underlying reasons for that: (i) different length-scales *l* for the processes where higher length-scales allow us to better estimate unobservable areas below obstacles; and (ii) various density of the obstacles within operating environment resulting in diversity of unreachable regions.

## 6. Experiments

The experiments are performed in order to verify that the system is operating successfully under laboratory conditions.

### 6.1. Experimental Setup

**Figure 14:**
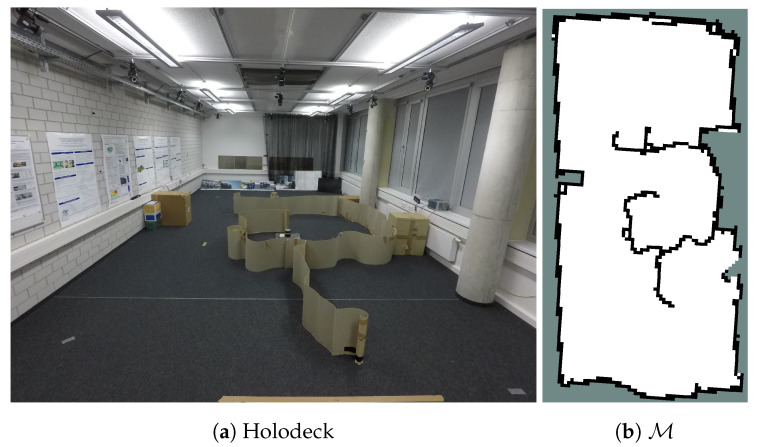
E1: Experiment with holonomic robotic platform.

The experiment (E1 in Table 1) is conducted with the slider (Figure 2a) in “Holodeck” laboratory, populated with obstacles (Figure 14a). All computations are carried out in real-time with the on-board computer of the robot. The aim is to reconstruct the magnetic intensity field of the “Holodeck”. As mentioned before (Section 4.1), we use real-world magnetic-field data with a simulated process sensor in the experiment.

First, the algorithm performance is tested with two different sampling distances for IE strategy:

- Finite horizon, r=[2,3] m.
- Sampling distance k=[2l,3l].

Additionally, IE with k=3l is compared against the aforementioned baselines (Section 5.3.1).

Robot operates in an environment of size $6\times 12m^{2}$, while the magnetic field data was collected within $4.6\times 9.6m^{2}$ (Table 1). Hyper-parameters of magnetic field intensity are $\theta ={\left[0.07,0.20,1.41\times 10^{-4}\right]}^{T}$ (Table 2). All runs start from $x^{\left[0\right]}={\left[0,0\right]}^{T}$ (the center of the operating environment).

As a remark, we can notice that in the real-experiment we have a bigger operating environment than process exploration area. This is due to a priori collected magnetic field measurements from partial environment. However, we allow path planner to plan through the whole operation environment while $X_{\mathrm{poi}}$ are generated only within the exploration area. This presents a possibility to collect data closer to exploration borders.

### 6.2. Experimental Results

As expected, based on previously obtained simulation results (Section 5.2), in order to reach the same NMSE with respect to the ground truth, a smaller sampling size results in (i) longer trajectories, i.e., longer exploration time, and (ii) more samples obtained, i.e., more computational costs (Table 8).

Real robot has its aforementioned physical limitations (here we set a safety radius of 0.6 m from the center of the robot) resulting in a higher NMSE for the explored process since we cannot measure in proximity and below obstacles. In evaluation, we assume that the process is fully explored when the NMSE is 0.34 with respect to the ground truth.

**Figure 15:**
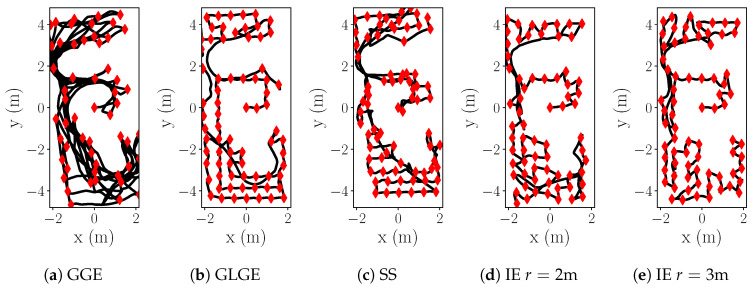
E1: Trajectories comparison, k=3l.

Based on the comparison of results presented in Table 9, we see that our systematic exploration (IE with horizon r=[2,3] m) results in significantly smaller distances needed for reaching NMSE 0.34. GGE (Figure 15a) strategy results in backward-forward motions. Both GGE and SS (Figure 15c) are suitable for fast map exploration, with the cost of notably slower process exploration. While GLGE (Figure 15b) behaves similarly with IE in narrow corridors, it lacks systematic approach within wider areas which produces longer trajectories due to returning to the vicinity of previously explored regions. We also see that with a small radius with respect to the rooms/corridors in the environment, our strategy (IE r=2 m, Figure 15d) can still miss a few important sampling locations and needs to traverse back. Nevertheless, both IE strategies (Figure 15d,e) result in shorter and more systematic trajectories.

**Figure 16:**
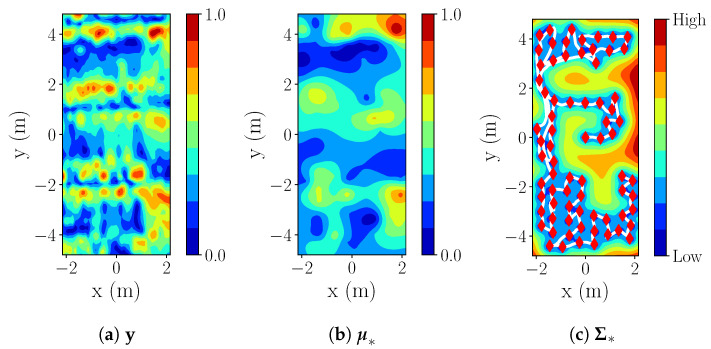
E1: IE, r=3m,k=3l with red diamonds in (c) representing measurements and white lines trajectory.

Figure 14b displays an example of the reconstructed occupancy grid map M and Figure 16b,c show the estimated process $y_{*}$ by the IE (r=3 m) in the real-world experiment for real magnetic field intensity of Figure 16a.

## 7. Conclusions and Future Work

This paper focuses on the exploration of a spatial process in an unknown environment with an autonomous mobile robot. More precisely, we address a scenario where two fundamental assumptions that are typically present in the state of the art do not hold: (i) A priori knowledge about the operating environment, and (ii) that process and map exploration can be solved independently. For addressing this, we propose an integrated strategy that solves the process and map exploration jointly by making the exploration strategy systematic. Meaning, it first explores local area before proceeding further towards unexplored areas.

Our approach has two essential components: (i) Selection of candidate points to decrease process uncertainty and increase map coverage, (ii) ordering of those points to minimize total travelled distance while incorporating knowledge about undiscovered map regions. We intertwine these two components by building on frontiers-based and GPs-entropy-driven exploration, A* path planner, and solutions to VRP.

We evaluated our integrated strategy in both simulations and experiments, using a simulated GP and magnetic field intensity as processes at hand, in various environments. The results allowed us to conclude the following: (i) Our strategy is able to decrease the error between process estimate (as calculated with GPs) and ground truth faster than GPs-greedy-entropy-based and frontiers-based strategies; (ii) trajectories resulting from our strategy follow a structured path, in contrast to the oscillatory behavior of GPs-entropy-driven exploration; and (iii) our integrated approach offers the best trade-off between process estimation error and map coverage.

To conclude, integrated exploration offers an opportunity to explore unknown environments efficiently, especially benefitting from segment exploration, e.g., exploring larger rooms or wide corridors in detail before moving towards “exits” to unknown space, controlled by the horizon parameter *r* and minimal sampling distance *k*. We note that expectantly choosing *k* closer to the length-scale *l* produces shorter trajectories, while sacrificing precision of process estimation.

Let us finally point out that in this work our focus was primarily on the development and testing of the exploration concept; exploitation of SLAM loop-closures was not in the scope of this paper. This is left for a future extension of the proposed system. Therefore, further efforts shall be devoted to a full integration of pose uncertainties propagated from SLAM into the GPs. Additionally, we aim to extend our exploration concept by incorporating an active SLAM component for an efficient trade-off between exploration–exploitation of both exploration and robot localization.

## Figures and Tables

**Table 1:** Evaluated scenarios in simulation (Sx) and Experimental (Ex) test cases.

Scenario	Process	Operating Environment	Dimensions $\left(m^{2}\right)$
S1	Process 1	Obstacle-free	8.0×8.0
S2	Process 1	Small room-like environment, obstacles introduced	8.0×8.0
S3	Process 2	Large room-like environment, obstacles introduced	56.1×38.4
E1	Magnetic field intensity	Obstacles introduced	12.0×6.0

**Table 2:** Explored GP process y with hyper-parameters θ.

Process	Dimensions ($m^{2}$)	$\sigma_{f}^{2}\left(m\right)$	*l* (m)	$\sigma_{n}^{2}$ (m)
Process 1	8.0×8.0	0.03	0.2	0.0001
Process 2	56.1×38.4	0.04	0.25	0.0001
Magnetic field	4.3×9.6	0.07	0.2	0.0001

**Table 3:** Performance evaluation for 2D field estimation with dataset with N=7592 measurements, *R* = 214,500 2D test points (390×550), grid resolution 0.1 m, hyperparameter l=0.25 m, sampling k=3l. We variate *P* and $p_{0}=N/P$ (number of local experts and their size, respectively).

$P,p_{o}$	Method	Prediction Time [s]	*NMSE*
37, 100	GRBCM	21.28	0.151
18, 200	GRBCM	20.74	0.093
12, 300	GRBCM	20.26	0.073
10, 350	GRBCM	19.26	0.070
9, 400	GRBCM	22.42	0.068
7, 500	GRBCM	24.31	0.062
1, 7592	Full GP	102.34	0.060

**Table 4:** Evaluated strategies compared against our Integrated Exploration (IE) strategy.

Strategy	Radius *r* (m)	Step Size *k* (m)	Multi-Step Planner
GGE	explored map	[3l, explored map]	No.
GLGE	(i) fixed *r*, (ii) explored map	(i) [3l,r], (ii) [3l, explored map]	No.
SS	explored map	(i) fixed = 3l, (ii) [3l, explored map]	No.
our IE	(i) fixed *r*, (ii) explored map	(i) [3l,r], (ii) [3l, explored map]	Yes.

**Table 5:** Mean value and standard deviation over five runs for scenario S1. Process exploration: Total travelled distance and number of collected process measurements ($N_{\mathrm{proc}}.$) required to achieve NMSE of 0.18 with respect to the ground truth.

Strategy	Distance (m)	$N_{\mathrm{proc}}.$
GGE	140.18±13.36	109±2
GLGE	107.06±2.12	134±3
IE r=2 m	102.82±3.51	134±3
IE r=3 m	105.39±1.84	134±2
IE r=full	104.49±2.79	138±4

**Table 6:** Mean value and standard deviation over five runs for scenario S2: (i) mapping: Total travelled distance needed to explore the map with accompanied number of obtained process measurements $N_{\mathrm{proc}.,\mathrm{map}}$ and process NMSE; (ii) process exploration: Total exploration distance needed to explore the process and number of collected measurements ($N_{\mathrm{proc}}.$) required to achieve NMSE of 0.21 with respect to the ground truth.

Strategy	Distance (m) Map Explored	$N_{\mathrm{Proc}.\mathrm{Map}}$	Proc. NMSE Map Explored	Distance (m) Proc. Explored	$N_{\mathrm{proc}}.$
GGE	15.84±4.38	13±5	0.6±0.1	126.31±5.13	84±1
GLGE	49.77±10.72	64±12	0.48±0.05	94.54±2.56	114±4
SS	25.72±6.63	35±9	0.62±0.02	108.17±7.38	118±4
IE r=2 m	69.94±12.58	89±15	0.42±0.07	89.89±4.97	114±2
IE r=3 m	64.89±17.68	85±17	0.47±0.12	90.97±3.96	116±2

**Table 7:** Scenario S3: (i) mapping: Total travelled distance needed to explore the map with accompanied number of obtained process measurements $N_{\mathrm{proc}.,\mathrm{map}}$ and process NMSE; (ii) process exploration: Total exploration distance to explore the process and number of collected measurements ($N_{\mathrm{proc}}.$) required to achieve NMSE of 0.084 with respect to the ground truth.

Strategy	Distance (m) Map Explored	$N_{\mathrm{Proc}.\mathrm{Map}}$	Proc. NMSE Map Explored	Distance (m) Proc. Explored	$N_{\mathrm{proc}}.$
GLGE	1398.45	2027	0.29	2977.52	3228
IE r=3 m	1393.46	1852	0.33	2847.06	3423
IE r=6 m	1888.78	2530	0.32	2872.52	3567

**Table 8:** Experiment E1—variation of sampling size: (i) mapping: Total travelled distance needed to explore the map with accompanied number of obtained process measurements up to that point $N_{\mathrm{proc}.,\mathrm{map}}$ and process NMSE; (ii) process exploration: Total exploration distance to explore the process and number of collected measurements ($N_{\mathrm{proc}}.$) required to achieve NMSE of 0.34 with respect to ground truth.

Strategy	Distance (m) Proc. Explored	$N_{\mathrm{proc}}.$	Distance (m) Proc. Explored	$N_{\mathrm{proc}}.$
	k=2l		k=3l	
IE r=2 m	92.84	144	55.63	75
IE r=3 m	93.56	146	58.23	74

**Table 9:** Experiment E1: (i) mapping: Total travelled distance needed to explore the map with accompanied number of obtained process measurements up to that point $N_{\mathrm{proc}.,\mathrm{map}}$ and process NMSE; (ii) process exploration: Total exploration distance to explore the process and number of collected measurements ($N_{\mathrm{proc}}.$) required to achieve NMSE of 0.34 with respect to ground truth.

Strategy	Distance (m) Map Explored	$N_{\mathrm{proc}.\mathrm{map}}$	Proc. NMSE Map Explored	Distance (m) Proc. Explored	$N_{\mathrm{proc}}.$
GGE	17.28	17	0.71	170.94	53
GLGE	44.91	54	0.62	64.81	75
SS	33.24	48	0.68	73.36	77
IE r=2 m	47.45	62	0.54	55.62	75
IE r=3 m	53.08	66	0.53	58.23	74

## Abbreviations

The following abbreviations are used in this manuscript:

GP	Gaussian process
NBC	nuclear, biological, chemical
SLAM	Simultaneous Localization and Mapping
BCM	Bayesian Committee Machine
RBCM	Robust Bayesian Committee Machine
TSP	Traveling Salesman Problem
FOV	field of view
GRBCM	Generalized Robust Bayesian Committee Machine
SE	squared exponential
LIDAR	Light Detection and Ranging
POI	Point of Interest
ICP	Iterative Closest Point
TEB	Time Elastic Band
WFD	Wavefront Frontier Detector
BFS	Breadth-first search
VRP	Vehicle Routing Problem
IE	Integrated exploration
NMSE	Normalized Mean Square Error
GGE	Greedy global entropy
GLGE	Greedy local-global entropy
SS	Sequential strategy
ROS	Robot Operating System
